# Natural expansion versus translocation in a previously human‐persecuted bird of prey

**DOI:** 10.1002/ece3.2896

**Published:** 2017-04-15

**Authors:** Virginia Morandini, Elena de Benito, Ian Newton, Miguel Ferrer

**Affiliations:** ^1^ Applied Ecology GroupDepartment of Ethology and Biodiversity ConservationEstación Biológica de Doñana (CSIC)SevillaSpain; ^2^ Centre for Ecology & HydrologyWallingfordUK

**Keywords:** *Aquila adalberti*, human disturbance, natal dispersal, refuges areas, reintroduction

## Abstract

Many threatened species in Europe have been expanding their distributions during recent decades owing to protection measures that overcome historical human activity that has limited their distributions. Range expansion has come about via two processes, natural expansion from existing range and reintroductions to new ranges. Reintroductions may prove to be a better way to establish populations because individuals are less subject to competitive relationships lowering breeding success than individuals expanding from existing populations. Whether this is true, however, remains uncertain. We compared success of breeding pairs of an expanding and a reintroduced population of spanish imperial eagles monitored for over 15 years in the south of Spain. We found significant differences in productivity between breeding pairs of each population. Newly established territories in reintroduction areas were almost three times more productive than new territories established as individuals expanded out from an existing population. We conclude that among these eagle populations reintroduced to new areas may fare as well or better than individuals expanding out form existing populations.

## 1 Introduction

Habitat loss and persecution has caused large predators to be largely confined to landscape locations that are subject to minimal human activity (Brown, McMorran, & Price, 2011; Chapron et al., 2014; Seddon, Griffiths, Soorae, & Armstrong, 2014). Changing human attitudes toward predators over recent decades (Pereira & Navarro, 2015) has led to growing concern over their fate to the extent that there are now widespread decreases in human persecution. Consequently, populations of predators are able to expand their ranges to recolonize areas from which they were previously extirpated (Chapron et al., 2014; Horváth et al., 2014). It also created opportunity to reintroduce predators to more distant locations that offer suitable habitat both (reinforcements and reintroductions) and outside of the historically indigenous range (assisted colonization) (Seddon et al., 2014). Natural range expansions and recolonization of vacant range are common in many species (Caniglia, Fabbri, Galaverni, Milanesi, & Randi, 2014; Gadenne, Cornulier, Eraud, Barbraud, & Barbraud, 2014; Kojola et al., 2006; Martin, Koeslag, Curtis, & Amar, 2014). Reintroductions involve the release of individuals into suitable vacant habitat where the species may or may not have been extirpated (Seddon, Armstrong, & Maloney, 2007).

It has been proposed, however, that individuals undergoing natural range expansion are constrained by density‐dependent reductions in mean productivity because they may be forced into poorer‐quality habitat by individuals holding established territories (Ferrer & Bisson, 2003; Ferrer & Donazar, 1996; Ferrer, Newton, & Casado, 2006, 2008; Korpimaki, 1988; Newton, 1998; Sergio & Newton, 2003). This comes about because in a low‐density population, individuals entering the breeding population are able to select optimal territories of high quality. As density increases, and the best territories become occupied, more and more individuals are forced to occupy poorer territories, where their reproductive success is lower. As the overall population increases, therefore, the mean breeding success (young per pair) falls. But, when individuals are released in a new area, lack of competition may allow individuals to occupy high‐quality territories allowing individuals to achieve a mean productivity higher than in the original population, and perhaps also breed at a younger age.

In well‐established populations, with greater competition, older individuals have more chance of occupying vacancies than young ones (Ferrer, Newton, & Pandolfi, 2009; Ferrer & Penteriani, 2003), and are more likely to occupy the better territories, so good reproduction is expected from adult pairs (Carrete, Sanchez‐zapata, Martinez, Sanchez, & Calvo, 2002; Carrete, Sánchez‐Zapata, Tella, Gil‐ Sánchez, & Moleón, 2006; Ferrer & Bisson, 2003). However, in low‐density situations, with young pairs occupying high‐quality territories, little difference in productivity between young and adult breeding pairs is expected (Ferrer & Bisson, 2003). In a natural colonization, with empty high‐quality habitat outside the old population boundaries, high productivity values are also expected for young pairs. These boundaries are promoted in philopatric species because of the tendency of individuals to return to the natal population to breed (Ferrer, 1993a). Expansion to areas just outside the boundaries of an existing population allows immature pairs to establish territories and start breeding at younger age than if they stayed within the existing population (Ferrer, Newton, & Muriel, 2013; Ferrer, Otalora, & GarcÍa‐Ruiz, 2004; González et al., 2006).

Here, we compared two different means of colonization in the Spanish Imperial eagle in order to test potential demographic differences: a natural expansion of a past restricted breeding population into new territories that have not been occupied for at least 30 years and a reintroduced population reintroduced into a new distant area that has not been occupied for at least 30 years.

## 2 Materials and Methods

### 2.1 Study sites and species

The Spanish imperial eagle is one of the rarest eagles in the world (Vulnerable in the IUCN Red List, BirdLife International 2008), with around 430 breeding pairs in 2014 (National Working Group, unpublished data 2014), located entirely in the Iberian Peninsula. The species is a large (2,500–3,500 g) long‐lived raptor, monogamous, sedentary and territorial, with a low annual productivity averaging 0.75 chicks/pair (Ferrer & Calderón, 1990). Reproduction usually lasts 8 months from February, when laying starts, until October when the latest juveniles leave the natal area (Ferrer, 2001). Independent juveniles disperse on “exploratory” movements (Ferrer, 1993a), using different temporary settlement areas (Ferrer, 1993b) but making periodic returns to their natal area where they are likely subsequently to breed. Individuals normally recruit to the breeding population at around 4–5 years old (but see Ferrer et al., 2004). Temporary settlement typically occurs in open lands that have high prey densities (especially wild rabbit *Oryctolagus cuniculus*), low human disturbance, and no other medium‐large breeding raptors (Ferrer & Harte, 1997).

Spanish imperial eagles can be divided into two easily distinguishable plumage classes: (1) subadult, with tawny‐colored plumage or dark patches over a tawny base, present until 4–5 years of age; and (2) adult, predominantly dark brown with characteristic white markings appearing from the age of 5 years (Ferrer & Calderón, 1990). The two age‐groups can be easily distinguished in the field.

The monitored nests were in Andalusia occupied a large part of the southern Iberian Peninsula and had a wide altitudinal range (0–2,000 m.a.s.l.), with a dry‐humid Mediterranean climate (annual rainfall: 300–2,000 mm, average annual temperature: 9–19°C). The landscape consisted of a mosaic of Mediterranean forests, scrublands, and grasslands in hilly and mountainous areas, crops in lowlands and coastal wetlands.

The fragmented distribution of existing populations of the Spanish Imperial eagle in Andalusia is the result of direct human persecution in the past (Mariano González et al., 2008), and the natural slow expansion of these populations into neighboring areas is more or less restricted to the edges of these refuges sites, regardless the quality of habitat available.

In the reintroduction project, the release site (in neighboring Cádiz Province) was selected for reasons of habitat suitability and potential connectivity with other Spanish Imperial eagle populations (González, Bustamante, & Hiraldo, 1992; Madero & Ferrer, 2002; Muriel, Ferrer, Casado, Madero, & Calabiug, 2011). It was situated 85 km away from the nearest established population in the Coto Donana (24 times the near neighbor distance in a high‐density population). The reintroduction project started in 2002 and continued until 2015, with a total of 87 chicks released by hacking being Sierra Morena the donor population.

### 2.2 Data analysis

**Figure 1:**
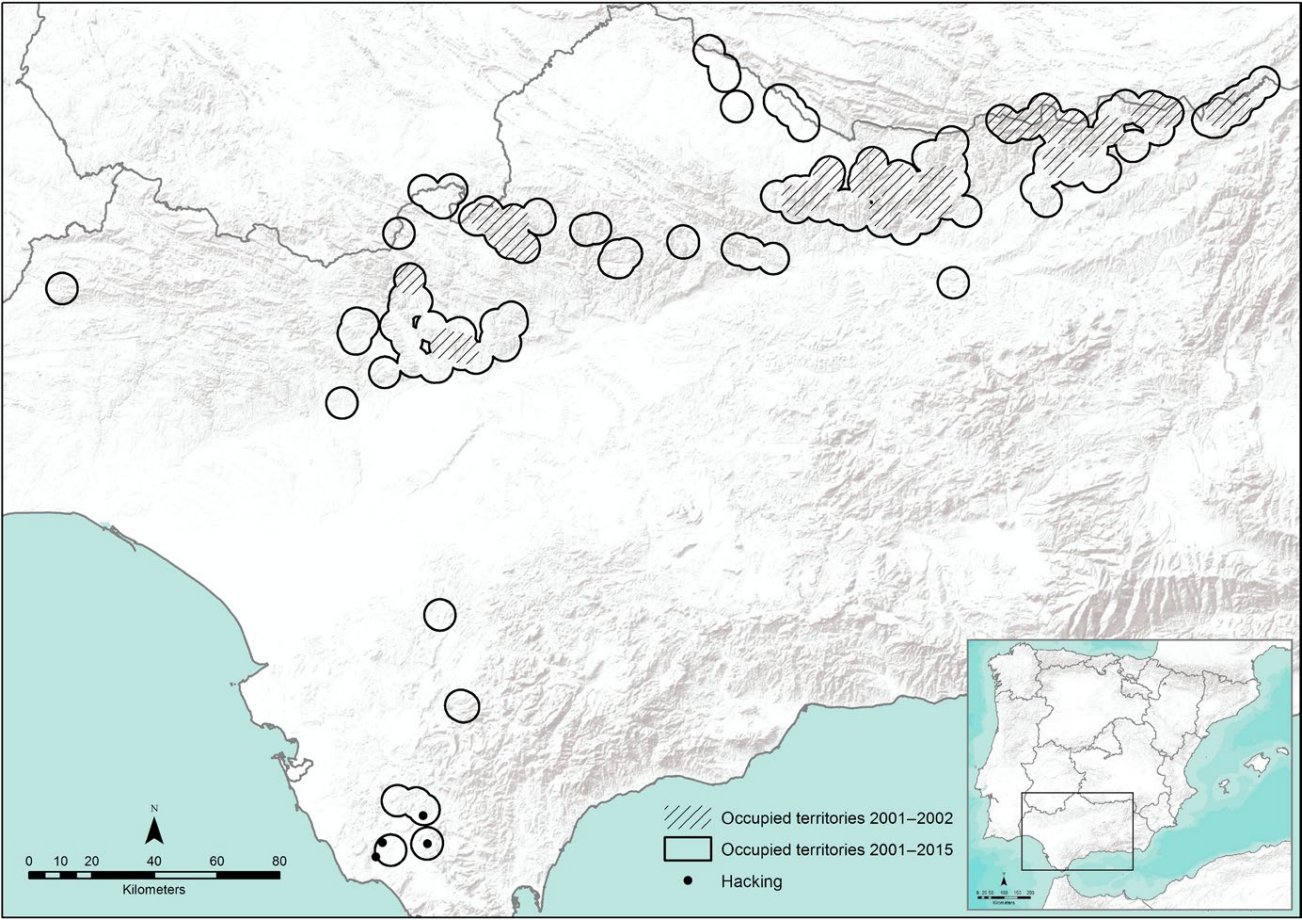
Distribution of Spanish Imperial eagle nests in Andalusia during the study period (2001–2015). Occupied territories during 2001–2002 are represented with shading lines. The expansion of the population is represented with a circle. The release points in the reintroduction area are the black spots in the Cádiz Province

During the study period, work was focused on two populations (see Figure 1), one occurring naturally in the Sierra Morena in the north limit of Andalusia (≈38°22′N 3°50′W) and the other reintroduced in Cádiz (≈36°20′N 5°48′W). Territorial pairs present in both populations were studied from 2001 to 2015. The data were derived from a total of 112 different territories and represented 763 breeding events. We considered a breeding event when a pair showed breeding behavior (nest construction, defense, incubation, etc.). All nests were monitored from the beginning of the breeding season (January–February, during courtship and nest site selection; Ferrer, 2001) until the last chick left the natal territory, and data on breeding, distance to nearest neighboring nest (NND), and the pair's plumage state were recorded. We refer to all pairs with at least one member in subadult plumage as “immature pairs.” Productivity was calculated as the number of fledglings per nest. To allow for annual variation in reproductive performance, we adjusted productivity (number of fledglings) for year effects by subtracting annual means from the raw data. Corrected data are referred to as relative values (Ferrer & Bisson, 2003; Horváth et al., 2014; Penteriani, Balbontin, & Ferrer, 2003). Also, as in other studies of raptors, territory quality was estimated by frequency of occupancy (Ferrer & Donazar, 1996; Newton, 1991; Sergio & Newton, 2003).

We distinguished between colonized territories and old territories inside the existing population accounting for both the location of the territory (with colonized territories established at locations in the external ring of nests in the existing population Ferrer, Belliure, Minguez, Casado, & Bildstein, 2014) and the NND. We used NND recorded in the Doñana population during 1993, a period of high density, with the maximum number of territorial pairs ever registered in the Doñana National Park. The mean neighbor nest distance was 3,464 m, ranging between 1,800 and 9,250 m (Ferrer, 1990). Consequently, we considered a peripheral area of 9,250 m around the existing population, considering as colonized territories all those with NND values higher than 9,250 m.

We distinguished between two different colonization types: (1) territories which appeared without human intervention in the peripheral limits of the Sierra Morena population (natural colonization, Sierra Morena) and (2) territories which appeared in the reintroduction area and with at least one member coming from the reintroduction project.

Finally, as other studies showed, body condition of nestling imperial eagles is closely related to hatching date (Ferrer, 1994; Muriel, Ferrer, Balbontín, Cabrera, & Calabuig, 2015). We tested for differences in nutritional conditions by analyzing differences in hatching dates between chicks from reintroduced and natural territories. For the analysis of the hatching dates, only nests from 2012 to 2015 (*N* = 216) had reliable hatching dates, expressed in relation to the earliest hatching recorded in the 3 years as Day 1 (Ferrer, 1994).

### 2.3 Statistical analysis

We calculated occupancy as the frequency of occupation of one territory from the first time that it was occupied to 2015. Also we excluded of this analysis territories with less of 3 years of occupation data. In order to check occupancy as measure of quality of territories, a Spearman correlation between occupancy and productivity (mean young per nesting attempt) was conducted.

To avoid potential pseudo‐replication due to the high potential for strong site‐fidelity and pair‐fidelity in this long‐lived species, a generalized linear mixed model (GLMM) was conducted with territories as a random effect. In this case, relative productivity was considered as the dependent variable over the years.

To remove any effect of age, we used a GLMM using only data from adult–adult pairs. To remove the effect of territory quality, we compared productivity parameters between immature and adult pairs present in the same territory with a nonparametric Wilcoxon signed‐rank test for pooled data for the 54 territories occupied in different years by adult‐immature pairs.

We used GLMM to test for differences in productivity among colonized and existing territories with territories as a random effect, and age of pair (adult plumage or immature plumage) and colonized or old territories as fixed factors. We tested differences between colonized territories from Sierra Morena and colonized territories from the reintroduction area. Knowing that the reintroduced population so far has no adult pairs occupying territories during our study period, a GLMM for colonized territories (both, natural and assisted) occupied only by immature pairs was used. Finally, hatching dates of chicks from the reintroduced and natural populations were compared.

## 3 Results

The number of breeding pairs of Spanish Imperial eagles in the south of Spain increased from 11 in 2001 to 91 in 2015. Annual population growth rate was calculated for the entire study period for each population as λ = 1.59 for the reintroduced population (one pair from the first territorial pair after the reintroduction project in 2010 to four pairs in 2015) and as λ = 1.17 for the naturally expanding population (10 pairs in 2001 and 87 pairs in 2015). Natural colonized territories appeared from the beginning of the study period with four territories with NNDs of more than 9,250 m and outside the external ring of nests of the existing population in 2001.

Occupancy varies from 100% (territory occupied every year since the first time it had been occupied) to 6.7% (territory occupied in only one breeding season during 2001–2015). Variation in territory occupancy was positively correlated with variation in mean standardized productivity (Spearman rank order correlations *N* = 81, Spearman *R* = .368, *p* = .0007). In other words, the most frequently occupied territories showed the highest average annual breeding success.

Analyzing all territories, differences in standardized productivity among territories were related to age of the pair and territory identity, pairs composed only by adult birds showed higher productivity than immature pairs (GLMM; age of pairs: *F* = 27.99, *df* = 1, *p* < .001; territory: *F* = 2.14, *df* = 101, *p* = .023). The highest value for relative productivity was found in territory number 4 (in the expanding population), occupied by an adult pair in 2001 and with a relative productivity of 2.56. The lowest value for relative productivity was found in territories 92 and 3 (both in the nucleus of the existing population), occupied by a young pair and an adult pair, respectively, with a relative productivity of −1.6 during 2001 and 2003.

To remove the effect of age class, we conducted analyses considering only data from adult–adult pairs. Again, productivity differed significantly among territories (GLMM; *N* = 523, territory: *F* = 2.373, *df* = 82, *p* < .001). Also, no differences were found in productivity between adult and immature pairs in the same territory (sign test. *N* = 54, *Z* = 1.497, *p* = .134). Significant differences in relative productivity between old territories and colonized ones (including both natural and assisted colonization) were found, resulting from the effect of age of the pair and territory identity, with both effects highly significant (Table 1). New colonized territories showed higher productivity levels than old ones (mean old territories: −0.049; colonized territories: 0.205).

**Table 1:** Results of the generalized linear mixed model of factors influencing relative productivity, including age of pair and type of population as a fixed effects and territory identity as a random factor

	Effect	*df* effect	MS effect	*df* error	MS error	F	*p*
1. Age of pair	Fixed	1	26.565	74.077	1.268	20.948	<.0001
2. Type of population	Fixed	1	10.713	75.605	2.182	4.908	.029
3. Territory identity	Random	96	1.833	14.011	0.692	2.646	.021
1 × 2	Fixed	1	0.124	31.271	0.727	0.170	.682
1 × 3	Random	46	0.741	523.000	0.811	0.913	.637

Significant terms were found in age of pairs, type of populations, and territory identity. Type of population: (1) existing population and (2) colonizing population (includes natural colonization and reintroduction).

Analysis of only colonized territories (natural and reintroduced) and comparison only of immature pairs (there were no adult pairs in the reintroduced population) revealed significant differences among territories and between the two colonization types (Table 2). Territories which appeared by reintroductions were 2.11 times more productive (mean relative productivity = 0.582) than those of natural colonization in the peripheral limits of the Sierra Morena population (mean relative productivity = −0.266).

**Table 2:** Results of the generalized linear mixed model of factors influencing relative productivity in immature pairs, including colonization type (reintroduction or natural colonization) as a fixed effect and territory identity as a random factor

	Effect	*df* effect	MS effect	*df* error	MS error	*F*	*p*
Colonization type	Fixed	1	7.259	17.871	1.563	4.641	.045
Territory identity	Random	24	1.392	45.000	0.812	1.714	.058

Colonization type emerged as a significant effect.

Finally, in immature breeding pairs, chicks from the reintroduced population show significant earlier hatching dates than chicks from all natural territories (GLMM; type of population: *F* = 44.823, *df* = 1, *p* < .005).

## 4 Discussion

During the twentieth century, most raptor populations in Europe declined, and their geographical ranges were reduced mainly by human persecution (Whitfield, 2004). However, nowadays most raptor species are increasing in both numbers and distributions (Ferrer et al., 2013; González et al., 2006; Horváth et al., 2014), and many species have been subject to reintroduction programs aimed to re‐establish them in former range. The present distribution of the Spanish Imperial eagle is mainly the result of past human persecution, so the current population mainly occupies remote and inaccessible areas that do not necessarily hold the best habitat for the species (Ferrer, Negro, Casado, Muriel, & Madero, 2007; González, Bustamante, & Hiraldo, 1990; González et al., 1992).

The Spanish Imperial eagle range expansion during the last decade was facilitated by a reduction of the number of electrocuted birds due to mitigation measures implemented on power poles (López‐López, Ferrer, Madero, Casado, & McGrady, 2011). This population growth was associated with the establishment of new territories in places that had not been occupied for at least 30 years (González et al., 1992; Horváth et al., 2014). It was to improve the species recovery that the reintroduction program started in Cádiz Province in 2002.

In a natural colonization, breeding pairs prefer to settle near existing ones and productivity is limited by the habitat quality present in those areas. In contrast, translocations are not relegated to areas surrounding existing populations and the selection of good habitats for the species is the main criteria in the choice of a release site (Armstrong & Seddon, 2008). For this reason, new territories limited to areas surrounding existing populations show lower productivity than territories in release areas selected by habitat quality values and without already established populations (Table 2). Immature pairs, whose productivity is highly dependent on the quality of the territory (Balbontín & Ferrer, 2008; Ferrer & Bisson, 2003; Ferrer et al., 2008), showed significantly higher productivity in the reintroduction area. Difference between productivity in Sierra Morena and Cádiz could be promoted only by differences in habitat quality being all the occupied territories in Cádiz territories of high quality for the specie. An alternatively explanation for the differences in productivity between natural and assisted colonization would be differences in quality of founder individuals of the reintroduction program. Genetic differences depending on the donor population would affect productivity. In our case, we can discard any genetic differences that could affect the productivity of breeding pairs because released individuals in the reintroduction area were translocated from the Sierra Morena population (Muriel et al., 2011).

As all breeding pairs in the Cádiz population contained at least one reintroduced individual fed ad libitum during the release process (Muriel et al., 2015), we cannot separate the effect of good physical condition in their first stage of life from the territory quality effect. If the higher productivity of the Cádiz population breeding pair was a consequence only of the ad libitum feeding of chicks, we expected a decrease in productivity in future breeding pairs without a reintroduced member. Nevertheless, body condition of nestling imperial eagles is closely related to hatching date (Ferrer, 1994; Muriel et al., 2015), with earlier hatching dates indicating better nutritional conditions. Muriel et al. (2015) established that released individuals in the reintroduction program were mainly later hatched birds in the season due to the extraction protocol during the program. Consequently, it seems that the idea of a priori better‐quality released birds could be discarded. Furthermore, significant differences in chick hatching dates between natural and reintroduced colonized populations support the idea of better territory quality in the reintroduction area.

As previous studies showed, the Spanish imperial eagle is a long‐lived species with slow turnover, strong philopatric behavior, and conspecific attraction (Ferrer, 1993a; González, 1989; Muriel, Morandini, Ferrer, & Balbontín, 2016). With these characteristics, reflected in a tendency to breed close to existing populations, areas without the presence of conspecifics or far away from established populations have little chance of being occupied. The main strategy of individuals trying to enter in a breeding population is to look for vacancies in the natal area during the beginning of the breeding season (Ferrer, Morandini, & Newton, 2015), as the presence of other breeding pairs apparently signals suitable habitat (Kivela et al., 2014).

The probability of getting a vacant territory inside an existing population is related to the time spent searching for vacancies (Ferrer & Penteriani, 2003), as well as experience and competitive ability (Balkiz et al., 2010), and knowledge of the locations and qualities of territories (Kokko, Harris, & Wanless, 2004). For these reasons, young individuals are less likely to find and fill vacancies in the natal area than older individuals, and breeding dispersal outside the population limits gives more chance of finding a potential territory. In fact, as our results show, during the process of population expansion, the number of immature pairs increases in territories with higher NND values (Ferrer et al., 2004, 2009, 2013; González et al., 1992; Horváth et al., 2014; Margalida et al., 2008), suggesting that immature pairs tend to establish territories far from existing nests. Where immature individuals are able to occupy high‐quality territories, values of productivity do not differ from adults in high‐quality territories, so productivity is not correlated only with the age of breeding pairs (Ferrer & Bisson, 2003; Ferrer et al., 2013; Horváth et al., 2014). We found no differences in productivity depending on age of breeding pairs in high‐quality territories. Our results confirm the importance of territory quality to productivity and the ability of young pairs to reproduce as well as adult pairs when they have the opportunity to occupy high‐quality territories (Ferrer & Bisson, 2003).

As other authors suggested, we found that occupancy of territories was related to productivity (Sergio & Newton, 2003). Expanding population processes show again that quality of territory has a major influence on the productivity of breeding pairs (Ferrer & Bisson, 2003; Ferrer & Donazar, 1996; Ferrer et al., 2013). The presence of empty habitat adjacent to existing population perimeters allows immature pairs to settle near their natal population (Horváth et al., 2014; Kivela et al., 2014), but reintroduction projects allow the occupation of vacant high‐quality habitat not limited by the existing population's distribution.

Territory quality seems to be a major driver of productivity (Osborne & Seddon, 2012) and even past refuges served well for the protection of target species in the era of human persecution. Now with a changing human attitude, the best habitats for the species may be empty and far away from existing population nuclei. Yet efforts to restore populations in their indigenous range tend to select release sites based on current habitat use under the assumption that the present location of the species represents optimal habitat. This could lead to a serious mismatch whereby suboptimal sites are chosen for reintroduction attempts, which subsequently fail to result in population establishment, growth, and persistence. The assessment of high‐quality habitat is a key step before starting a reintroduction project.

## Conflict of Interest

None declared.

## Data Accessibility

All Spanish Imperial eagle data used in this manuscript (productivity, nest location, individual plumage) are available from Consejería de Medio Ambiente (Junta de Andalucía).
